# Overcoming Shellfish Allergy: How Far Have We Come? †

**DOI:** 10.3390/ijms21062234

**Published:** 2020-03-23

**Authors:** Christine Y.Y. Wai, Nicki Y.H. Leung, Ka Hou Chu, Patrick S.C. Leung, Agnes S.Y. Leung, Gary W.K. Wong, Ting Fan Leung

**Affiliations:** 1 Department of Paediatrics, The Chinese University of Hong Kong, Prince of Wales Hospital, Shatin, Hong Kong; 2 Hong Kong Hub of Paediatric Excellence, The Chinese University of Hong Kong, Shatin, Hong Kong; 3 School of Life Sciences, The Chinese University of Hong Kong, Shatin, Hong Kong; kahouchu@cuhk.edu.hk; 4 Division of Rheumatology/Allergy, School of Medicine, University of California, Davis, CA 95616, USA; psleung@ucdavis.edu

**Keywords:** allergen-specific immunotherapy, component-resolved diagnosis, diagnosis, DNA vaccine, tropomyosin

## Abstract

Shellfish allergy caused by undesirable immunological responses upon ingestion of crustaceans and mollusks is a common cause of food allergy, especially in the Asia-Pacific region. While the prevalence of shellfish allergy is increasing, the mainstay of clinical diagnosis for these patients includes extract-based skin prick test and specific IgE measurement while clinical management consists of food avoidance and as-needed use of adrenaline autoinjector should they develop severe allergic reactions. Such a standard of care is unsatisfactory to both patients and healthcare practitioners. There is a pressing need to introduce more specific diagnostic methods, as well as effective and safe therapies for patients with shellfish allergy. Knowledge gained on the identifications and defining the immuno-molecular features of different shellfish allergens over the past two decades have gradually translated into the design of new diagnostic and treatment options for shellfish allergy. In this review, we will discuss the epidemiology, the molecular identification of shellfish allergens, recent progress in various diagnostic methods, as well as current development in immunotherapeutic approaches including the use of unmodified allergens, hypoallergens, immunoregulatory peptides and DNA vaccines for the prevention and treatment of shellfish allergy. The prospect of a “cure “for shellfish allergy is within reach.

## 1. Background

Shellfish is one of the most common food allergens, and allergy to shellfish usually persists throughout life. This is different from allergies to hen’s eggs and cow’s milk to which children often gradually acquire natural tolerance [1]. IgE-mediated allergic reactions induced by shellfish involve single or multiple organs and vary from immediate reactions to late-phase reactions up to 8 h after food consumption. Oral symptoms with mouth and throat itching and lip swelling are the most frequently self-reported symptoms. Oral food challenge-related reactions are followed by cutaneous reactions such as urticaria, periorbital angioedema and skin redness [2]. Food is the most common cause of anaphylaxis that requires presentation to Emergency departments in both the Asian and Western countries. Food represents the top cause of anaphylaxis at 29.9% in the United States with shellfish being the third most common food eliciting anaphylactic reactions (16.1%) following peanut (23.1%) and tree nuts (21.6%) [3]. When only adult subjects were evaluated, shellfish became the predominant anaphylaxis-eliciting food (34.4%). Similarly, in Singapore, Thailand, and Hong Kong, a majority of the food-induced anaphylactic cases were attributed to shellfish at frequencies of 24.6%, 45.2% and 71.2%, respectively [4,5,6]. Social impacts of food avoidance, dietary limitation, and reluctance to carry an epinephrine auto-injector (EAI) often have negative effects on the quality of life of the affected subjects [7]. Our survey of the local families in Hong Kong with affected children also revealed substantial adverse impacts of food allergy on parents’ quality of life, although allergy to shellfish was less detrimental when compared to peanut and egg [8].

### 1.1. Epidemiology

**Figure 1:**
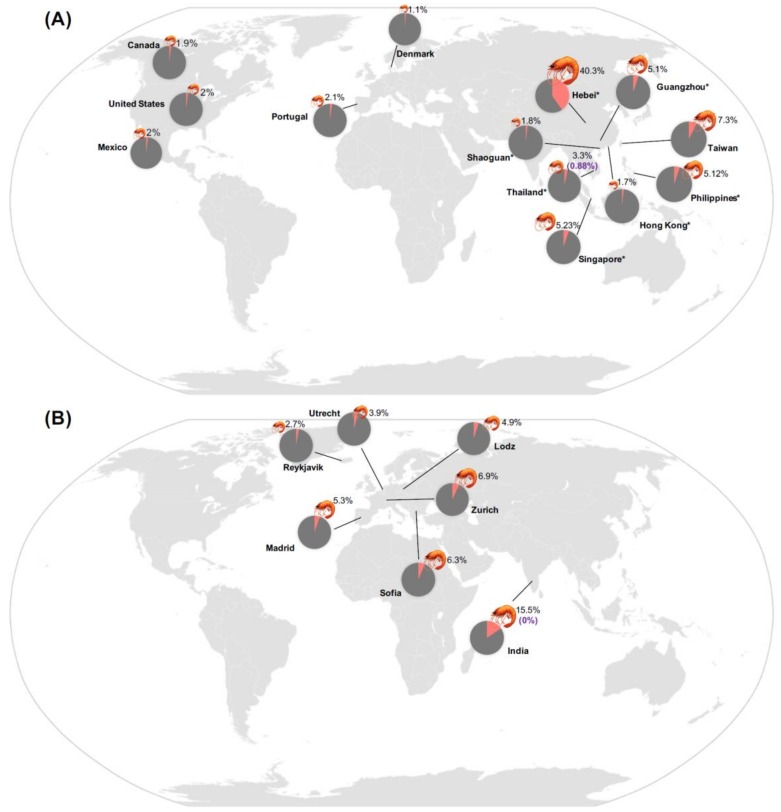
**Prevalence of shellfish allergy.** The estimated rate of shellfish allergy (pink) in populations at different geographical areas based on (**A**) self-reports and (**B**) IgE sensitization. Percentage in purple indicates the prevalence of shellfish allergy based on oral food challenge results. Asterisk (*) indicates that only children and/or adolescent populations aged 18 years or below were included in the estimation of shellfish allergy prevalence.

Shellfish allergy affects up to 10.3% of the general population, thus posing a growing public health issue worldwide [9,10]. The exact prevalence rates depend on the method of diagnosis, geographic areas and consumption habits. Based on a large-scale telephone survey, the prevalence of self-reported shellfish allergy was 2.0% among 14,949 Americans (Figure 1) [11]. The percentage was higher in adults (2.5%) than in children (0.5%). A more recent analysis based on the electronic health record (EHR) in the United Sates reported shellfish as the most common food allergen group (0.9%), followed by peanut (0.5%) and tree nut (0.4%) [12]. Another telephone survey in Canada revealed the prevalence of shellfish allergy to be 1.9%, which was the leading type of food allergy regardless of education and income levels [13]. Shrimp (1.3%) and other shellfish (0.7%) triggered a majority of hypersensitivity reactions among all surveyed food types in Mexican schoolchildren [14].

In Europe, a multi-center survey reported 4.8% of adults to have IgE sensitization to shrimp as compared to hazelnut (9.3%), peach (7.9%) and apple (6.5%) [15]. Shellfish was the leading food allergen in Portugal, affecting 34.6% of all food-allergic patients and 2.1% of the entire study population of 840 adults [16]. Most children from the Isle of Wight birth cohort reported shellfish allergy during adolescence, by which none of these subjects had IgE sensitization to shellfish at 1, 2, and 4 years while 0.2% of the subjects developed shellfish allergy at 10 and 18 years [17]. Such a trend for shellfish allergy was opposite to that of cow’s milk and hen’s egg allergies. Similar patterns were observed in a Danish study [18]. Among 597 children aged ≤ 3 years, none of them were sensitized to shrimp or with oral food challenge-confirmed shrimp allergy. Possible sensitization and confirmed allergy to shrimp were only found in subjects aged 3 years or older and were most frequent among the adult population. Out of the 121-adult food allergic subjects, 10 were found with both history of self-reported shrimp allergy and positive sensitization to shrimp, while three were confirmed of shrimp allergy by oral food challenge.

The prevalence of shellfish allergy is generally higher among Asians. A questionnaire-based survey in Hebei Province of China revealed the respective prevalence rates of allergies to crab, shrimp and shellfish to be 15.3%, 13.9% and 11.1% among 459 children and adults with rhinitis and/or asthma [19]. Shellfish was also the top food allergen (7.3%) among Taiwanese children and adults [20]. Among 3,677 Hong Kong preschoolers and 7,393 children aged below 15 years, the prevalence of parent-reported adverse reactions to shellfish was 1.3% and 1.7%, respectively [21,22]. Among schoolchildren in southern China, the prevalence rate of doctor-diagnosed crustacean allergy was significantly higher in urbanized Guangzhou (5.1%) than those in the rural Shaoguan (1.8%) [23]. When defined by reported symptoms and positive skin prick test/specific IgE (≥ 0.7 kU/L), shrimp remained as the leading offender in Hong Kong (1.05%), Guangzhou (0.18%) and Shaoguan (0.65%) [24]. In Singapore and the Philippines, 5.2% and 5.1% of teenagers reported a convincing allergic history after shellfish ingestion. These rates were higher than those of peanut (0.47% and 0.43%) and tree nut (0.3% and 0.33%) allergies [25].

Most epidemiological studies reported allergy to crustaceans (shrimp, crab and lobster) but not mollusks. Besides, most reports from Asia-Pacific focused on children and adolescents but not adults. The EuroPrevall-INCO study in India on the discrepant rates of IgE sensitization and clinical allergy to shrimp show that while 15.5% of subjects were sensitized, none had probable shrimp allergy [26]. This observation can be partly supported by the strong associations for IgE sensitizations among shrimp, house dust mite (HDM) and cockroach (i.e., cross-reactivity) [23,26]. Another study from Thailand found that only 0.9% children reacted to oral food challenges whereas 3.3% of children were reported by their parents to have adverse reactions to crustaceans [27]. These findings agreed with the common clinical observations that allergy sufferers often attributed their adverse food reactions to food allergy. In such cases, the addition of diagnostic tests such as skin prick tests or specific IgE assays would rule out a substantial proportion of self-reported food allergy and cross-reactivity.

### 1.2. Cross-Reactivity among Shellfish Allergens

The shellfish types causing clinical allergy are, in decreasing incidence, shrimp, crab, lobster, clam, oyster and mussel [28]. The muscle protein tropomyosin is the major cross-reactive allergen [29], which is a coiled-coil structured and heat-stable protein containing 276–284 amino acid residues with a molecular weight of 34–38 kDa (Table 1). Shrimp tropomyosins are the most well-studied shellfish allergens, which have been cloned as recombinant allergens such as *Pen a* 1 from *Penaeus aztecus* [30,31]. Sensitization to tropomyosin ranged from 23% to 83% of the subjects depending on the geographic populations while IgE against whole shrimp extract was present in 94% of subjects [31,32], suggesting that there are other minor shellfish allergens (Table 1). The 40-kDa arginine kinase (AK) and 20-kDa sarcoplasmic calcium binding protein (SCP) accounted for 10–15% of all sensitizations, [33] whereas the 20-kDa myosin light chain (MLC) was reported in >50% of shrimp-allergic subjects [34]. Interestingly, 74% of children and 10% of adults with shrimp allergy recognized SCP respectively, implying that this minor shellfish allergen is however a major trigger in the pediatric population [35]. A subgroup of shrimp-allergic children with MLC-specific IgE reported asthmatic episodes following exposure to steam from boiling shrimp [34]. More recently defined shellfish allergens, namely troponin C (TnC, a 20 kDa muscle protein), triose-phosphate isomerase (TIM, a 28 kDa glycolytic enzyme) and fatty-acid-binding protein (FABP, a 15–20 kDa transport protein) accounted for the hypersensitivity in 10–23% of shellfish-allergic patients [36,37]. Despite technological advances, many potential shellfish allergens as listed in Table 1 remain poorly characterized [38]. Most well-defined shrimp allergens are of low molecular weight, except hemocyanin, which is a 60–80 kDa protein accounting for 29–47% of IgE sensitization [33,39]. Moreover, Pascal et al. reported common co-sensitization to tropomyosin with MLC or SCP (36% and 31%, respectively) in a multi-center study in USA, Brazil and Spain [40], while co-sensitization to tropomyosin with AK or SCP was only observed in 5% of Italian patients [33]. A more recent pilot study, however, reported higher sensitization rate of TnC (50.0%) compared to tropomyosin (42.1%) among 74 shellfish allergic subjects in Hong Kong. Co-sensitization to AK and FABP was also common in this cohort [41]. These observations underpin the complex IgE reactivity profiles and population-specific sensitization patterns attributable to climatic and dietary factors. It is also important to highlight that these “minor” allergens were identified from shrimp and less commonly from crab species, whereas tropomyosin remains as the only allergen reported in other crustaceans and mollusks.

Tropomyosin is highly conserved among arthropods (e.g., crustaceans, house dust mites (HDM), cockroach) and mollusks, with an average homology of 61% shared among the two big groups of cross-reactive tropomyosins [42]. Specific IgE level of 4.38 kU_A_/L to rPen a 1 had a positive likelihood ratio of 10.9 for diagnosing mollusk allergy [43]. Of special interest is the high amino acid homology of 81% among tropomyosins from crustaceans, HDM and cockroach, which possibly explains aero-sensitization by tropomyosins from HDM and/or cockroach as a cause of shellfish allergy in Asia where HDM sensitization is prevalent [44,45]. Despite the assay-proven cross-reactivity of AK among shrimp, lobster, crab and crawfish [46], no clinical data have been established on cross-reactivity regarding SCP and MLC despite their high sequence homology. Out of 15 shrimp-allergic subjects, 13 failed double-blind placebo-controlled food challenges (DBPCFC) with mealworm (*Tenebrio molitor*) which is increasingly consumed in America and Europe [47]. Interestingly, oral challenges confirmed selective allergy to the giant freshwater shrimp (*Macrobrachium rosenbergii*) and the marine tiger shrimp *Penaeus monodon* [48]. Species-specific shellfish allergy might be attributed to the low sequence homology between allergens of the caridean and penaeid shrimp (to which the above two species belong, respectively) as well as by the presence of species-specific allergen such as hemocyanin [39]. These findings highlight the unmet needs of accurate shellfish allergy diagnosis due to non-cross-reactive specific allergens.

### 1.3. Diagnosis of Shellfish Allergy

Similar to other food allergies, the standard diagnostic approach for shellfish allergy involves a thorough review of clinical history [49] followed by in vivo skin prick test (SPT) and/or in vitro serum specific IgE (sIgE) measurement. Test results are then evaluated to determine the need for an oral food challenge. In this regard, we have learnt from the increasing number of reports concerning the sensitivity, specificity and perhaps the shortcomings of these conventional procedures [50]. In the study by Gámez et al. [51], 45 subjects were classified into shrimp-allergic, shrimp-tolerant and dust-mite groups according to DBPCFC results. Although all 18 shrimp-allergic cases had positive SPTs and sIgE to shrimp extract, 61% and 55% of shrimp-tolerant subjects and 11% and 44% of dust-mite patients also had positive SPT and sIgE results, respectively. These results suggest low specificity (approximately 50%) of these conventional diagnostic tests for shellfish allergy.

Apart from cross-reactivity among shellfish, HDM and cockroach, the predictive values of SPT and sIgE measurement against shellfish extracts could also be hampered by the variable qualities of allergen extracts. Comparison of freshly-prepared extracts from five commercial crustaceans revealed dramatic loss of protein bands in some preparations [52], resulting in heterogeneous SPT sensitivity of 59–79% among the subjects depending on the choice of test extracts. On the other hand, DBPCFC is the gold standard for food allergy diagnosis and is highly reliable. It can be applied in food allergy studies by masking the food in chocolate pudding or burgers minced with chicken meat and herbs [40,53]. However, the resource-intensive, time-consuming and expensive nature of this procedure as well as reluctance of subjects due to fear of side effects are critical barriers to implementing DBPCFC in the clinical setting.

It would thus be preferable to have an improved surrogate test to circumvent the drawbacks of these conventional diagnostic tests. Component-resolved diagnosis (CRD) that involves the detection of sIgE to individual allergenic molecules and/or allergen peptides has emerged to possibly resolve these ambiguities. In the same study by Gámez et al. [51], measurement of sIgE to r*Pen a* 1 showed improved specificity of 77% compared to extract-based SPT and sIgE analysis. However, Pascel et al. reported a specificity of only 56% for measuring sIgE to tropomyosin [40]. In contrast, positive sIgE to SCP yielded 94% specificity and higher positive likelihood ratio (LH + ) of 5.5 when compared to tropomyosin and shrimp extract (1.9 and 1.3, respectively). Sensitization to MLC was more frequent among food challenge-positive subjects. Shrimp AK and hemocyanin, on the other hand, were interpreted as cross-reactive markers as both were recognized by 75% of patients who were atopic to HDM and/or cockroach.

Furthermore, Pascel et al. synthesized overlapping peptides of shrimp tropomyosin, AK, MLC, SCP and TnC to determine the diagnostic values of IgE-binding and IgG_4_-binding peptides. Epitopes of tropomyosin had up to 100% specificity but variable sensitivity of 33–86% for shrimp allergy. In particular, tropomyosin epitope region p51–55 (KEVDRLEDELVNEKEKYKSITDELDQTFSELSGY) yielded the highest area under the curve (AUC) value, while epitope p29–30 (MDALENQLKEARFLAEEADR) had the highest LH+ value of 7.62 in comparison to shrimp extract, allergens and allergen peptides. Upon validation, this peptide-based CRD approach might be more specific for diagnosing shrimp allergy.

Quantification of sIgE to shellfish extract or allergens neither measures the capacity of these proteins to crosslink IgE nor provides information on IgE-mediated hypersensitivity reaction. Both the basophil activation test (BAT) and the emerging IgE-crosslinking-induced luciferase expression (EXiLE) test might offer solutions to these issues. The latter assay made use of a rat basophilic leukemia cell line transfected with human IgE receptor FcεRI and luciferase reporter gene (RS-ATL8) [54], which obviates the need for complicated procedures involving blood stimulation and flow cytometry-based analyses while keeping the benefits of measuring specific allergen-IgE crosslinking. A very recent study by Jarupalee et al. [55] presented remarkable findings, where only the 38 kDa- and 115 kDa-shrimp protein fractions induced higher reporter signals in EXiLE test while fractions containing shrimp proteins of 19 kDa, 38 kDa, 41 kDa and 115 kDa displayed IgE reactivity in Western blot. The 115-kDa proteins indeed exhibited weak IgE reactivity but were capable of inducing sufficient IgE crosslinking. However, further investigations involving more DBPCFC-confirmed shellfish allergic subjects with highly purified native or recombinant shellfish allergens via reporter assay are essential to conclude the utility of such IgE crosslinking-based assay for shellfish allergy diagnosis.

### 1.4. Shellfish-Specific Immunotherapy

In spite of the high prevalence of shellfish allergy, active treatment options are not yet available. It is recommended to shellfish allergic subjects that they avoid shellfish that trigger symptoms, to take antihistamines to alleviate mild symptoms, and to be treated with adrenaline, corticosteroid and β_2_-agonist in cases of anaphylactic reactions. Allergen-specific immunotherapy (AIT) represents a novel strategy to desensitize these food-allergic patients and restore their food tolerance. Although the effectiveness of oral (OIT), sublingual (SLIT) and epicutaneous (EPIT) immunotherapy for peanut, cow’s milk and egg allergies has been demonstrated [56], these emerging treatments have not been tested in patients with shellfish allergy. Clinical management of shellfish allergy remains challenging. The delineation of molecular characteristics of shellfish allergens over the past decade paves the way for the design of different immunotherapeutic strategies to treat this prevalent allergic disease.

### 1.5. Shrimp Extract and Allergens

The current AIT paradigm involves the administration of gradually increasing amounts of the allergen extracts or the recombinant allergens in an attempt to induce desensitization, and more preferentially, tolerance. The use of shellfish extracts or allergens in AIT for shellfish allergy is currently not in clinical use, and relevant published studies are scarce. In Egypt, the efficacy of sublingual shrimp immunotherapy was evaluated in 60 patients with shrimp allergy and 20 healthy controls, who received daily sublingual shrimp allergen extracts at increasing amount [57]. Six months later, significant reductions in shrimp-specific IgE and eosinophil count, accompanied with significant increases in IgG levels were detected in all patients. However, this study did not provide details on the use of a placebo control, end-point food challenge results, safety of the therapy or the changes at the T cell level.

Our group recently investigated the dose-dependent effect and safety of recombinant tropomyosin *Met e* 1 in a murine model of shrimp hypersensitivity [58]. BALB/c mice induced with shrimp allergy were treated thrice by low (0.01 mg), medium (0.05 mg) or high (0.1 mg) r*Met e* 1 through the intraperitoneal route. All groups of treated mice were successfully desensitized with significant decrease in specific IgE levels, although only the low- and medium-dose groups had signs of regulatory response. Despite the fact that low-dose immunotherapy was well tolerated, medium-dose AIT triggered mild allergic symptoms during the treatment phase, severe anaphylactic responses (convulsion and tremor) or even death were observed in the high-dose group. This might be attributed to the well-spaced epitopes and exceptional IgE crosslinking capacity of tropomyosin [59,60]. These observations indicate that recombinant shellfish tropomyosin in AIT should be used with much caution.

### 1.6. Hypoallergens

**Figure 2:**
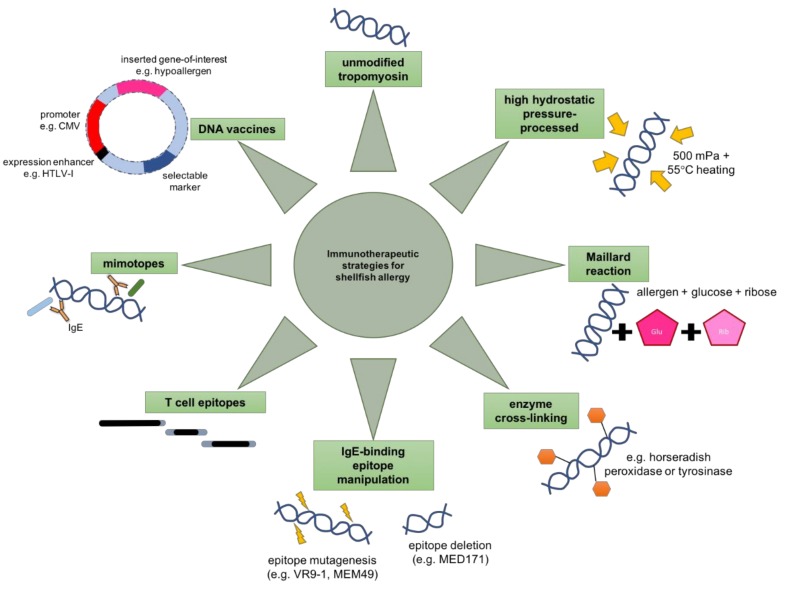
**Immunotherapeutic strategies for overcoming shellfish allergy.** The applicability of unmodified tropomyosin as a sublingual immunotherapy (SLIT), hypoallergens constructed by high hydrostatic pressure processing, Maillard reaction, enzyme cross-linking and epitope manipulation, immunoregulatory peptides including T cell epitopes and mimotopes, as well as hypoallergen-encoding DNA vaccines has been investigated over the past years.

Specific immunotherapy using the causal allergen is the conventional treatment option for most food allergies, but adverse events during treatment are likely, as shown by our group [58]. Modifying recombinant allergens to reduce their IgE reactivity and allergenicity is a core strategy in improving the safety of AIT. Over the past years, several strategies including high hydrostatic pressure processing, chemical modification, polypeptide fragmentation and IgE-binding epitope manipulation have been attempted to engineer hypoallergenic tropomyosins (Figure 2).

### 1.7. High Hydrostatic Pressure Processing

High hydrostatic pressure (HHP) has been applied in the food industry to inactivate microorganisms and enzymes while preserving the flavor, color and nutrient content of the food [61]. By combining HHP at 500 MPa and heating at 55°C for 10 min, shrimp tropomyosin extracts exhibited almost complete loss of IgE reactivity as assessed by inhibition ELISA with mouse sera [62]. Interestingly, higher pressure at 600 MPa increased IgE reactivity of the extract again. On the other hand, the delivery of HHP-treated tropomyosin to BALB/c mice sensitized to purified tropomyosin resulted in the reduction of serological levels of specific IgE and histamine, as well as downregulation of *IL-4* in the small intestine compared to mice given the unmodified tropomyosin extract. However, HHP-treated tropomyosin also down-modulated *IFN-γ* and *IL-10* expression, thus casting doubt on its immuno-modulatory function.

### 1.8. Chemical Modification

A general approach adaptable to constructing hypoallergens of different food allergens is desirable. Methods such as absorption to polyphenols and fusion of allergen to mannoside or flagellin were successful in engineering hypoallergenic food proteins [63,64,65]. Maillard reaction with glucose and ribose was also shown to be effective in reducing the allergenicity of the major cherry allergen *Pru av* 1 [66]. When such technology was applied on squid tropomyosin, a clear and significant reduction in its allergenicity was confirmed by dot-blotting and competitive inhibition ELISA [67]. Rather than altering the α-helix content or the structure of the allergen, the Maillard reaction lowered the digestibility of squid tropomyosin. However, the IgE-binding capacity of scallop tropomyosin was, on the contrary, enhanced by Maillard reaction with ribose [68]. Therefore, this strategy might not be widely applicable to reduce the allergenicity of shellfish tropomyosins.

On the other hand, Liu et al. [69] attempted the cross-linking of crab tropomyosin to horseradish peroxidase or tyrosinase to produce CHP and CTC, respectively. Comparatively, the reduction of IgE-binding activity of CHP was more prominent than CTC, as suggested by the >60% reduction in IgE binding in ELISA and histamine release in RBL degranulation assay. Such an effect was postulated to be due to significant reduction in the α-helix content and/or the increased digestibility upon enzyme cross-linking. When CTC or CHP were used to sensitize mice, lower levels of crab tropomyosin-specific IgE and IgG_1_ but higher level of IgG_2a_ were found when compared to mice sensitized to unmodified tropomyosin. Although CHP appeared to have lower allergenicity, its capacity in inducing IgG_2a_ antibody was significantly lower than that by CTC. Mice sensitized to CTC or CHP also produced less IL-4 and IL-13 but more IFN-γ and a higher frequency of Foxp3^+^ Treg cells in the spleen. Thus, CTC and CHP preferentially induced Th1- and/or Treg-oriented immune response *in vivo*. Additionally, the group employed the same strategy and demonstrated that cross-linking arginine kinase from crab with horseradish peroxidase or tyrosinase also significantly reduced its allergenicity and promoted its capacity in inducing oral tolerance in mice [70]. Clinical trials regarding the safety and efficacies of these enzyme cross-linked allergens in treating crab allergy are imminent.

### 1.9. Polypeptide Fragmentation

Another logical concept of engineering hypoallergens is to limit the structural features of an allergen in activating the IgE receptor FcεRI. Mahajan et al. recently designed five sequential *Pen a* 1 recombinant polypeptides and dissected their capacities for inducing histamine release in basophils and RBL cells that exclusively express the human IgE receptor FcεRIα subunit (hRBL^r^^αKO^ cells) [59]. Polypeptides are continuous and longer peptide bonds with more than fifty monomer units. Specifically, in this study, five overlapping polypeptide fragments with 60–79 residues in length derived from *Pen a* 1 were recombinantly produced (PP1 - PP5 at positions 1–70, 68–127, 121–181, 172–236 and 224–284 respectively). Each polypeptide retained at least one major IgE-binding and T cell epitopes. The secretory responses of hRBL^r^^αKO^ cells triggered by individual or pooled polypeptides were reduced comparing to that triggered by *Pen a* 1 even at the highest tested concentration (1,000 ng/mL). These polypeptides could also out-compete *Pen a* 1 and inhibit mediator release from hRBL^r^^αKO^ cells, suggesting reduced allergenicity of these truncated polypeptides and their potential for immunotherapeutic evaluation. Yet, the basophil histamine release patterns triggered by each polypeptide were unique among different allergic subjects, which might imply the need for tailored-design or further optimization of these polypeptides.

### 1.10. Epitope Manipulation

Manipulation of the immunodominant IgE-binding epitopes represents a sophisticated strategy to precisely dampen the IgE reactivity and allergenicity of an allergen. The first attempt to construct hypoallergenic tropomyosin deploying this concept was reported by Reese et al. [71] upon defining the eight IgE-binding epitopes within the five major IgE-reactive regions of *Pen a* 1. [72] Based on their combinatorial amino acid substitution analysis, a *Pen a* 1 mutant VR9-1 was engineered by introducing 12 mutations within the major epitopes. The allergenic potency of VR9-1 showed a reduction of 10- to 40-fold comparing to *Pen a* 1 in the RBL histamine release assay. However, the maximal releases of this mutant were comparable to that of *Pen a* 1, suggesting that the substitutions were not sufficient to fully abolish the IgE reactivity of *Pen a* 1 and/or the missing-out of important IgE-binding epitopes.

To circumvent this issue, three new regions on the shrimp tropomyosin *Pen m* 1 with lengths of 14–18 amino acid residues (peptide 1 at positions 23–40; peptide 4 at positions 115–128 and peptide 8 at positions 210–224) were predicted to be the linear epitopes by means of immuno-informatics analyses [73]. Their IgE reactivity was confirmed by dot-blot inhibition experiment, by which these newly identified epitopes could inhibit specific IgE from binding to tropomyosin at levels comparable to other known *Pen a* 1 epitopes. Subsequently, by means of an array of immuno-assays and immunoinformatics tools, our group further refined the identity of IgE-binding epitopes in shrimp tropomyosin (*Met e* 1) and identified an additional IgE-reactive region (epitope E7 at positions 236–241) [74].

Collectively, nine major IgE binding epitopes of shrimp tropomyosin with 5–21 amino acid residues were found that supported the subsequent construction of new hypoallergenic variants of shrimp tropomyosin. Our group designed two hypoallergens of shrimp tropomyosin, namely MEM49 and MED171, by substituting the IgE-binding epitopes of *Met e* 1 with the homologous non-allergenic tropomyosin sequences of four edible fish species (Atlantic salmon, orange-spotted grouper, Mandarin fish and Atlantic bluefin tuna), as well as by deleting all the nine IgE-binding epitopes, respectively [74]. The in vitro reduction of IgE reactivity of MEM49 and MED171 were 71.4% and 77.4% relative to native *Met e* 1, respectively, when reacting to sera of shrimp-allergic subjects. MEM49 and MED171 also displayed significantly reduced in vivo allergenicity in a passive cutaneous anaphylaxis assay.

The more important finding from this study was the induction of inhibitory IgG antibody by MEM49 and MED171. Serum IgG antibody from MEM49- or MED171-immunized BALB/c mice blocked 46.2% and 45.9% IgE of shrimp-allergic subjects from binding to native *Met e* 1. In this context, successful immunotherapy is usually associated with increased IgG_4_ antibody with inhibitory functions [75]. This type of antibody may intercept the interaction between IgE and the allergen, decreasing antigen presentation by down-modulating CD23-mediated antigen uptake and/or suppressing IgE-mediated degranulation by mast cells and basophils upon IgG/FcγRIIB and IgE/FcεRI co-aggregation [76,77,78,79]. The immunotherapeutic values of MEM49 and MED171 are thus highlighted by their capacity to induce IgG with validated inhibitory functions.

### 1.11. Immunoregulatory Peptides

Another mainstream AIT approach deploys allergen-derived T cell peptides or mimicry synthetic peptides to modify inflammatory responses (Figure 2). Accordingly, the T cell epitopes of the shrimp tropomyosin *Pen a* 1 have been identified using peripheral blood mononuclear cells (PBMCs) from shrimp-allergic patients [80]. Validated by both the tritiated-thymidine incorporated proliferation and cytokine release assays, a total of 17 tropomyosin peptides containing the allele-restricted T cell epitopes were identified. Subsequently, our group identified six 20-mer peptides, T1 to T6, at *Met e* 1 positions 26–45, 56–75, 86–105, 146–165, 221–240 and 251–270, respectively, from the proliferation and cytokine release responses of splenocytes from BALB/c with *Met e* 1-induced shrimp allergy [81]. These epitopes nested within the T cell epitopes of *Pen a* 1, suggesting a conserved set of immunogenic sequences between shrimp-allergic subjects and the BALB/c murine model.

In the same study, our group also validated the immunotherapeutic potency of the identified T cell epitopes. Delivery of the T cell epitope mixture as an oral immunotherapy to *Met e* 1-sensitized BALB/c mice significantly reduced the symptom scores, serological levels of specific-IgE and mMCP-1, improved diarrhea and infiltration of inflammatory cells (i.e., goblet cells and eosinophils) to the intestinal tract. This peptide oral immunotherapy also induced the synthesis of IgG_2a_ antibody with confirmed inhibitory functions. Although limited by the lack of flow cytometric analysis precisely showing the expansion of Treg cells population, expression of *IL-10, FOXP3, CD25* and *RUNX1* were all upregulated in the small intestine of peptide-treated mice, indicating the induction of regulatory responses by this treatment. It is noteworthy that treatment using six non-T cell epitope peptides of *Met e* 1 neither led to the above-mentioned resolution of allergic responses nor the induction of local regulatory responses, thus emphasizing the immunoregulatory values of these six T cell peptides. Further analyses of shorter peptides with more overlapping sequences in human T cell lines are needed to refine the T cell epitopes of *Met e* 1.

Other than the allergen-derived T cell epitopes, mimotopes are also potent immunoregulatory peptides for therapeutic application. Mimotopes are short peptides resembling the epitopes of an allergen that can induce epitope-specific antibodies [82,83,84]. The monovalent property of mimotopes can also prevent the cross-linking of IgE and the subsequent mast cell degranulation, thus enhancing the safety of AIT. Through scanning the one-bead-one-compound peptide libraries against IgE of shrimp allergic subjects, our group identified 25 mimotopes of shrimp tropomyosin [85]. Six mimotopes with the highest-ranking scores were selected for in vivo validation. BALB/c mice immunized with the individual mimotope conjugated to KLH produced *Met e* 1-recogonzing IgG antibodies, and such response was not observed in mice immunized with KLH only or mimotope of the HDM allergen *Der p* 1. Future studies on the immunotherapeutic capacity of these *Met e* 1 mimotopes will be intriguing [86].

### 1.12. DNA Vaccine

DNA vaccine-based immunotherapy is another emerging trend in the treatment of allergies [87,88], as it has many advantages over traditional protein-based vaccines. The process requires only the cloning of the gene-of-interest (e.g., the causal allergen, hypoallergens and/or peptides) into a plasmid vector that thus substantially reduce the production cost. A proposed mechanism of antigen presentation by DNA vaccines is the attraction of professional antigen presenting cells (e.g., dendritic cells) to the site of injection, followed by on-site transfection, expression and presentation of the antigen by these cells to prime both the CD4 and CD8 T cells [89]. The in vivo expression of the cloned gene is driven by the eukaryotic promotor over much longer periods of time as compared to protein antigens [90]. The DNA vaccine thereby better primes the immune system to offer long term immune memory and potentially support tolerance induction via promoting Treg cell responses [91,92]. Moreover, the built-in CpG motifs in the DNA vaccine backbone activate toll-like receptor 9 (TLR9) that leads to the activation of MyD88-dependent signaling pathways which drive Th1-oriented immune responses [93]. Such Th1- and tolerance-promoting properties thus make DNA vaccines a logical approach to confront allergic diseases.

**Figure 3:**
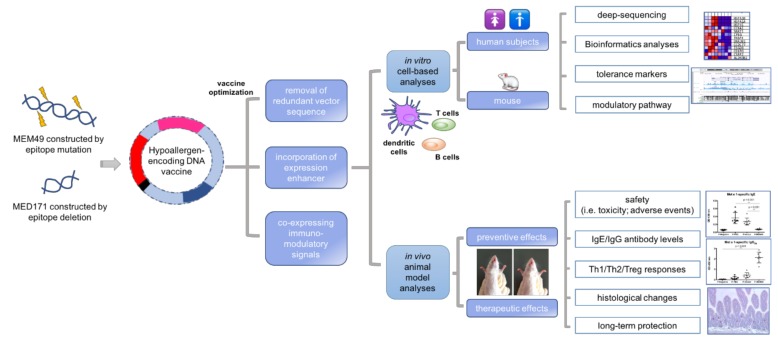
**Experimental design of the hypoallergen-encoding DNA vaccine study.** Two hypoallergens, MEM49 and MED171, of shrimp tropomyosin were constructed by epitope mutation and deletion, respectively. DNA vaccines were then constructed by cloning and expressing the hypoallergens in mammalian expression plasmids. Our data demonstrated that pMEM49 and pMED171 were effective in down-modulating shrimp tropomyosin-induced allergy in BALB/c mice, such as suppressing the *Met e* 1*-*specific IgE and inflammatory responses in the gut while increasing specific IgG_2a_ with inhibitory functions. Optimization of the vaccines would follow in constructing next-generation DNA vaccines with enhanced in vivo expression of the hypoallergen and immunogenicity of the vaccines. In vitro cell-based analyses involving the major immune players (e.g., dendritic cells, T and B cells) in human and mouse to dissect the modulatory mechanism of the hypoallergen-encoding DNA vaccines, as well as in vivo analyses in murine model for the safety and therapeutic efficacies of the vaccines are essential in addressing the applicability of the vaccines for the clinical treatment of shellfish allergy.

In light of this, our group has focused on investigating the applicability of hypoallergen-encoding DNA vaccines to prevent and treat tropomyosin-induced shrimp allergy [94]. In our first step, we cloned the tropomyosin hypoallergens, MEM49 or MED171, into the mammalian expression vector pCI-Neo to construct two DNA vaccine candidates (pMEM49 and pMED171; Figure 3). BALB/c mice with shrimp tropomyosin-induced hypersensitivity treated with pMEM49 or pMED171 had their allergic symptoms ameliorated significantly [95]. This was in line with the significant reduction in levels of specific IgE and expression of Th2 cytokines, as well as infiltration of inflammatory cells in the small intestine (i.e., mast cells and eosinophils). Such treatment also greatly enhanced both the local and systemic regulatory responses, but to a greater extent by pMED171 treatment. Our group also further demonstrated the functionality of the pMED171-induced Treg cells in limiting intestinal inflammation and Th2 immune activities via anti-CD25-mediated depletion and adoptive transfer experiments, as well as the capacity of pMEM49 in inducing gene signatures of Treg cell and tolerogenic dendritic cells activities, thereby implicating the clinical values of these hypoallergen-allergen encoding DNA vaccines against shellfish allergy [96].

## 2. Conclusions

In cow’s milk, egg and peanut allergies, there is extensive literature on the clinical efficacies of different OIT, SLIT and EPIT regimens [50,97], as well as novel pioneering studies on diagnostic and AIT strategies. The FAST project aiming at testing hypoallergens against fish and peach allergies is actively underway [98]. On the other hand, there is paucity of data regarding the diagnosis and immunotherapy of shellfish allergy. The cross-reactivity of tropomyosins among arthropods and the clinical contribution of shellfish allergens other than tropomyosin add complexity to the precise diagnosis and design of AIT for shellfish allergy. We note that diagnosis could not rely on one single test. While CRD and IgE-crosslinking assays hold the promise of improving diagnostic accuracy, large-scale studies in populations with high prevalence of shellfish allergy are lacking. sIgE cut-off levels for defining a shellfish allergy, and strategies to identify non-IgE mediated late-phase allergic reactions triggered by shellfish are also highly anticipated. The drawbacks of conventional AIT, including too-frequent and extended clinical visits to complete an AIT, recurrence of allergy after discontinuation of treatment and unresponsiveness to AIT present additional barriers in formulating shellfish allergy-specific immunotherapy. Although the novel AIT approaches discussed herein are still at the experimental stages, they provide exciting and promising strategies for clinical trials of AIT for shellfish allergy. Specifically, human applications of DNA vaccines have lagged and the use of this technology in treating food allergy is still in infancy, in part due to the suboptimal expression and immunogenicity of DNA vaccines in human and, on the other hand, the under-defined immuno-modulatory mechanism of this approach. Yet this is likely to be just a matter of time and future reports on next-generation DNA vaccines for shellfish allergy treatment are envisaged.

## Figures and Tables

**Table 1:** List of identified shellfish allergens.

Allergen	Allergen Nomenclature	Molecular Weight (kDa)	Rate of IgE Sensitization	Route of Exposure	Cross-Reactivity
Tropomyosin	*Lit v* 1; *Met e* 1; *Pen a* 1; *Pen m* 1; *Pen b* 1	34–38	23–83%	Ingestion	Arthropods (crustaceans, HDM, cockroach), mollusks
				Inhalation	
Arginine kinase	*Lit v* 2; *Pen m* 2	40–45	10–15%	Ingestion	Shrimp, lobster, crab and crawfish
				Inhalation	
Sarcoplasmic calcium-binding protein	*Lit v* 4; *Pen m* 4	20–25	10–15%	Ingestion	None
Myosin light chain	*Lit v* 3; *Pen m* 3	17–20	>50%	Ingestion	None
Troponin C	*Cra c* 6	21–21	20–30%	Ingestion	NR
Triose phosphate isomerase	*Cra c* 8; *Pen m* 8	28	20–30%	Ingestion	NR
				Inhalation	
Fatty acid-binding protein	-	15–20	10.3%	NR	NR
Hemocyanin	-	60–80	29–47%	NR	*Macrobrachium resenbergii, Penaeus monodon* and HDM
Paramyosins	-	100	NR	NR	Mollusks
myosin heavy chain	-	225	NR	NR	NR
𝛼-actin	-	94–99	NR	NR	Shrimp and HDM
β-actin	-	41–46	NR	NR	Shrimp and HDM
ubiquitin	-	5–8.5	NR	NR	Shrimp and HDM
Glyceraldehyde phosphate dehydrogenase	-	37	NR	NR	NR
Smooth endoplasmic reticulum Ca ^+ +^ ATPase	-	113	NR	NR	NR

NR: not reported.

## Patents

US 15173467: Mimotopes for use in immunotherapy for shellfish and/or arthropod allergy. US 10301365: Met e 1 tropomyosin variants for use in allergen-specific immunotherapy.
